# Insertion of a knockout-first cassette in *Ampd1* gene leads to neonatal death by disruption of neighboring genes expression

**DOI:** 10.1038/srep35970

**Published:** 2016-10-24

**Authors:** Yongcheng Pan, Lusi Zhang, Qiong Liu, Ying Li, Hui Guo, Yu Peng, Hexiang Peng, Beisha Tang, Zhengmao Hu, Jingping Zhao, Kun Xia, Jia-Da Li

**Affiliations:** 1 Key laboratory of Hunan Province in Neurodegenerative Disorders, Xiangya Hospital, Central South University, Changsha, Hunan, China; 2 Department of Ophthalmology, the Second Xiangya Hospital, Central South University, Changsha, Hunan, China; 3 The State Key Laboratory of Medical Genetics and School of Life Sciences, Central South University, Changsha, Hunan, China; 4 Department of Neurology, Xiangya Hospital, Central South University, Changsha, Hunan, China; 5 Mental Health Institute of the Second Xiangya Hospital, Key Laboratory of Psychiatry and Mental Health of Hunan Province, Central South University, Changsha, Hunan, China; 6 College of Life Science and Technology, Xinjiang University, Xinjiang, China; 7 Collaborative Innovation Center for Genetics and Development, Shanghai, China

## Abstract

AMPD1 is an adenosine monophosphate deaminase that catalyzes the deamination of AMP to IMP. To understand the physiological function of AMPD1, we obtained a strain of *Ampd1* mutant mice from KOMP repository, which was generated by a knockout-first strategy. An elevated AMP level and almost complete lack of IMP was detected in the skeletal muscle of E18.5 *Ampd1*^*tm1a/tm1a*^ mice. However, *Ampd1*^*tm1a/tm1a*^ mice died in 2 days postnatally, which was contradicting to previous reports. After removal of the knockout-first cassette and critical exon, mice homozygous for the *Ampd1*^*tm1c/tm1c*^ and *Ampd1*^*tm1d/tm1d*^ alleles survived to adulthood. RNA-seq analysis indicated that the expression of two neighboring genes, *Man1a2* and *Nras*, were disrupted in the *Ampd1*^*tm1a/tm1a*^ mice, but normal in the *Ampd1*^*tm1c/tm1c*^ and *Ampd1*^*tm1d/tm1d*^ mice. The neonatal lethality phenotype in the *Ampd1*^*tm1a/tm1a*^ mice was consistent with the *Man1a2*-deficient mice. Our results indicated the knockout-first cassette may cause off-target effect by influence the expression of neighboring genes. This study, together with other reports, strongly suggests that removal of targeting cassette by site-specific recombinases is very important for the accurate phenotypic interpretation on mice generated by target mutations.

Mice with artificial disruption in a gene have been widely applied to understand the gene function *in vivo*. With the accomplishment of human and mouse genome projects, a variety of strategies have been employed to generate genome-wide mutagenesis or knockout. Recently, Bradley and colleagues report a knockout-first strategy to generate mutant alleles for thousands of genes in mice. The knockout-first combines the advantage of both a reporter-tagged and a conditional mutation^1^. As shown in Fig. 1a, a cassette containing mouse En2 splicing acceptor (SA), LacZ, and promoter-driven neomycin resistant gene (Neo) is inserted in introns of target genes. The initial allele (tm1a) is predicted to generate a null allele through splicing to the LacZ trapping element. Conditional alleles (tm1c, Fig. 1b) can be generated by removal of the gene trap cassette using Flp recombinase. The tm1d allele (Fig. 1c) is conventional mutation after crossing tm1c allele with Cre transgenic mice. This multi-purpose strategy is applied to European Conditional Mouse Mutagenesis (EUCOMM) and the National Institutes of Health Knockout Mouse programs (KOMP) to generate knockout mice, which are available for the researchers world-wide.

Adenosine monophosphate (AMP) deaminase (AMPD, EC 3.5.4.6) is a group of enzymes that catalyze the deamination of AMP to IMP. AMPD family contains three members, AMPD1-3^2^. AMPD1 is mainly expressed in the skeletal muscle, whereas AMPD2 is widely expressed in non-muscle tissues and AMPD3 is mainly expressed in erythrocytes^3^,^4^. AMPD is the initial reaction of the purine nucleotide cycle, which plays a central role in the adenine nucleotides metabolism and energy charge maintenance^5^. Thus AMPD is thought to play an important role in regulating nucleotide metabolism, as well as maintaining adenylate energy charge. Several studies have reported that AMPD1 deficiency might regulate muscle metabolism^6^,^7^. Polymorphism in *AMPD1* gene has been reported to be associated with metabolic myopathy, which is also named as AMP deaminase deficiency^8^,^9^. The disease is reported an approximate 2–3% incidence in the Caucasian population, as well as a similar prevalence in African-Americans^10^. In our previous study, a haplotype *AMPD1-NRAS-CSDE1* is found to be associated with autism^11^, and variants in *AMPD1* contribute to autism risk in Chinese Han population^12^.

In this study, we studied a strain of *Ampd1* mutant mice generated by a knockout-first strategy. Our data indicated that insertion of knockout-first cassette into *Ampd1* gene disrupted the expression of neighboring genes up to 2.4 Mb, which may underlie their neonatal death.

## Results

### *Ampd1* ^*tm1a/tm1a*^ mice died in 2 days postnatally

To study the physiological function of AMPD1, we obtained a strain of *Ampd1* mutant mice from KOMP repository, which was generated by the knockout-first strategy. The tm1a allele was initially a non-expressive form due to the alternative splicing of the *Ampd1* exon1-2 to SA in the trapping cassette, disrupting the *Ampd1* transcript. As shown in Fig. 2, *Ampd1* was predominantly expressed in the muscle as determined with real-time PCR (RT-PCR) and Western blot (Fig. 2a,b); no *Ampd1* mRNA and protein were detected in the hind-leg muscle of E18.5 *Ampd1*^*tm1a/tm1a*^ mice (Fig. 2c,d).

When intercrossing the *Ampd1*^*tm1a/*+^ mice, the progeny at embryonic 18.5 days (E18.5), postnatal 0.5 days (P0.5) and P1 showed a Mendelian distribution, but no *Ampd1*^*tm1a/tm1a*^ pups survived more than two days (Table 1), indicating *Ampd1*^*tm1a/tm1a*^ mice may die within 24–48 hours postnatally. No apparent histopathological anomaly except lung was found in the P0.5 *Ampd1*^*tm1a/tm1a*^ mice. As shown in Fig. 3a, *Ampd1*^*tm1a/tm1a*^ mice showed volume reduced pulmonary alveoli and incrassated alveolar septum compared to wide-type (WT) mice.

The *Ampd1*^*tm1a/tm1a*^ pups could be easily recognized by lack of milk in their stomachs (Fig. 3b). Moreover, the body weight of *Ampd1*^*tm1a/tm1a*^ pups at P0.5 was significantly lower than that of WT mice and *Ampd1*^*tm1a/*+^ mice (Fig. 3c). The *Ampd1*^*tm1a/tm1a*^ mice at P0.5 also showed significantly decreased blood glucose compared with heterozygous and WT mice (Fig. 3d), implying starvation in neonatal *Ampd1*^*tm1a/tm1a*^ mice.

### Impaired nucleotide metabolism in the muscle of *Ampd1* ^*tm1a/tm1a*^ mice

AMPD1 catalyzes AMP hydrolyzing into IMP and NH_3_, and plays an important role in the purine nucleotide cycle^5^. We therefore measured the nucleotide level in the tissues from *Ampd1*^*tm1a/tm1a*^ and their WT and *Ampd1*^*tm1a/*+^ controls. A dramatically elevated AMP level (over 200%) and almost complete lack of IMP were detected in the skeletal muscle of E18.5 *Ampd1*^*tm1a/tm1a*^ mice (Fig. 3e), indicating that AMPD1 was the major, if not solely, adenosine monophosphate deaminase in the skeletal muscle. Consistent with the restricted expression of *Ampd1* in the muscle, there was no genotypic difference in the nucleotide levels of the brain, heart, liver and lung (Supplementary Fig. 1).

### Disruption in neighboring genes expression in *Ampd1* ^*tm1a/tm1a*^ mice

Another two strains of *Ampd1*-deficient mice showed similar deficiency in the purine nucleotide cycle as *Ampd1*^*tm1a/tm1a*^ mice; however, they were viable and did not show significant difference in life span^13^,^14^. We thus speculated an off-target effect in our strain may underlie the phenotypic difference.

We then analyzed the global gene expression in the skeletal muscle from E18.5 animals by using RNA-seq. In addition to the deficiency in *Ampd1*, we also identified another two down-regulated genes, *Man1a2* and *Nras* (Supplementary Table 1); RT-PCR analysis indicated that *Man1a2* and *Nras* expression in the muscle of *Ampd1*^*tm1a/tm1a*^ mice was reduced to 24% and 51% of WT mice, respectively, The expression of *Man1a2* were also dramatically decreased in other tissues, such as the brain, heart, kidney and lung (Fig. 4a), whereas the expression of *Nras* was slightly reduced in the liver (Fig. 4b). It should be noted that *Man1a2*-deficient mice have been reported died at postnatal 0.5 day with pulmonary hypoplasia, hypercellular, thicker alveolar septae, and alveolar hemorrhage^15^.

### The knockout-first cassette influences neighboring genes expression

Interestingly, *Man1a2, Nras* and *Ampd1* are all located on chromosome 3. *Nras* is 6 kb from *Ampd1* gene, whereas *Man1a2* is 2.4 Mb away from *Ampd1* (Fig. 4c). Nevertheless, the expression of genes located between *Man1a2* and *Ampd1* did not change in the muscle and lung (Supplementary Table 1 and Supplementary Fig. 2). To see if down-regulation of *Man1a2 and Nras* was caused by the insertion of knockout-first cassette or the deficiency of AMPD1 protein *per se*, we removed the FRT-flanked knockout-first cassette by crossing *Ampd1*^*tm1a/*+^ with a globe Flp transgenic mouse strain to produce tm1c allele. When intercrossing *Ampd1*^*tm1c/*+^ mice, the progeny showed a Mendelian distribution and *Ampd1*^*tm1c/tm1c*^ mice were viable in adulthood (Table 1). The *Ampd1*^*tm1c/tm1c*^ mice showed normal gene expression for *Man1a2, Nras* and *Ampd1* in the skeletal muscle (Fig. 4d).

We then mated *Ampd1*^*tm1c/tm1c*^ with a globe Cre transgenic mouse strain to produce tm1d allele. When intercrossing *Ampd1*^*tm1d/*+^ mice, the progeny showed a Mendelian distribution and *Ampd1*^*tm1d/tm1d*^ mice were viable in adulthood (Table 1). Although *Ampd1* was disrupted in the *Ampd1*^*tm1d/tm1d*^ mice, the expression of *Man1a2* and *Nras* in the skeletal muscle of *Ampd1*^*tm1d/tm1d*^ mice was indistinguishable from those of WT controls (Fig. 4d). Our data thus indicated that it was the knockout-first cassette, instead of the deficiency of AMPD1 protein, influenced the expression of *Man1a2* and *Nras*.

## Discussion

It is widely accepted that insertion of a selection marker such as PGK-neo in an allele may disrupt neighboring gene expression. Targeted mutation of several genes, including β-globin, Granzyme B, Hox genes, and MRF4, by insertion of PGK-neo selection cassette results in severe reduction of neighboring gene expression up to 100 kb away from the mutation sites^16^,^17^,^18^,^19^,^20^,^21^. Furthermore, FRT/loxP sites used in gene targeting could also influence the expression of neighboring genes^22^.

Insertion of a knockout-first cassette has also recently been reported to influence neighboring genes expression. Maguire *et al*. find that homozygous *Slc25a21*^*tm1a*^ exhibit orofacial abnormalities, alterations in carpal and rugae structures, hearing impairment and inflammation in the middle ear. However, after removal of the critical exon and knockout-first cassette, *Slc25a21* knockout mice homozygous for the *Slc25a21*^*tm1c*^ and *Slc25a21*^*tm1d*^ alleles are phenotypically indistinguishable from wild-type. RNA expression analysis indicate that the expression of *Pax9*, located at the 3′ end of *Slc25a21* on the opposite strand with 188 bp overlapping at the 3′-UTR regions, is reduced specifically in mice homozygous in tm1a, but not in tm1c and tm1d alleles.

There are several explanations for this phenomenon. One possibility is that a cluster of genes share one regulatory element, and targeting cassette disrupted the interaction between corresponding genes and the upstream regulatory element. For instance, the insertion of PGK-Neo into granzyme B genes, located at the most 5′ of granzyme gene cluster, severely reduces the normal expression of a panel of genes within the locus up to 100 kb away from the mutation^17^. In contrast, a targeted mutation of the promyelocyte-specific cathepsin G gene, which lies just 3′ to the granzyme genes in the same cluster, has minimal effects on upstream granzyme gene expression. However, our case seems much more complicated. Although insertion of the knockout-first cassette in *Ampd1* influences *Man1a2* and *Nras* expression, more than twenty genes localized between *Man1a2* and *Nras* are essentially unaffected.

Another probable explanation is that the targeting cassette disrupted the chromatin conformation-dependent interactions between genes and their distant regulatory elements. A commonly accepted chromatin loop model is that DNA sequences between regulatory elements and promoters form loops to allow the distant regulatory elements to interact directly with the promoters. Chromosome conformation capture (3C) and 3C-derived methods^23^,^24^,^25^,^26^,^27^ have demonstrated chromosome three-dimensional conformation in a genome-wide level, which is hosted in the WashU Epigenome Browser^28^. Interestingly, there are indeed long range interactions between *Ampd1* and *Man1a2*^29^ (Supplementary Fig. 3). We thus speculate that insertion of knockout-first cassette may disrupt some of these interaction, and subsequently downregulate the expression of *Man1a2*.

The *Man1a2* null mice die within 12 h after birth because of respiratory distress and some delay in the lung development^15^. The *Ampd1*^*tm1a/tm1a*^ mice also show deficiency in the lung development, consistent with the downregulation of *Man1a2*. However, *Ampd1*^*tm1a/tm1a*^ mice die within 24–48 hours postnatally, probably due to starvation. Furthermore, Tremblay *et al*. also report the loss of L-PHA, E-PHA, and DSA reactive glycoproteins in a subpopulation of epithelial cells lining the bronchioles from *Man1a2* null mice; however, there is no difference in these lectin-stained glycoproteins in the *Ampd1*^*tm1a/tm1a*^ mice (data not shown). We speculate the residual expression of *Man1a2* (i.e. 24% in the muscle and 43% in the lung) may underlie the phenotypic difference between *Man1a2* null and *Ampd1*^*tm1a/tm1a*^ mice. Nevertheless, we cannot exclude the possible contribution of other genes deficiency to the neonatal lethality of *Ampd1*^*tm1a/tm1a*^ mice.

In summary, insertion of knockout-first cassette *per se* in *Ampd1* gene results in globally reduced expression of neighboring gene *Man1a2*, which may partly explain the neonatal lethality phenotype of *Ampd1*^*tm1a/tm1a*^ mice. Our results indicate the knockout-first cassette may cause off-target effect by disruption the chromatin conformation-dependent interaction between genes and their distant regulatory elements and influence the neighboring gene expression. We thus strongly suggest users to remove the knockout-first cassette by Flp recombinase, and then generate conditional allele or global allele using specific Cre recombinases. In addition, high throughput assays, such as RNA-seq, may be used to verify if there is any side-effect in the initial allele.

## Methods

### *Ampd1* knockout mice

**Figure 1:**
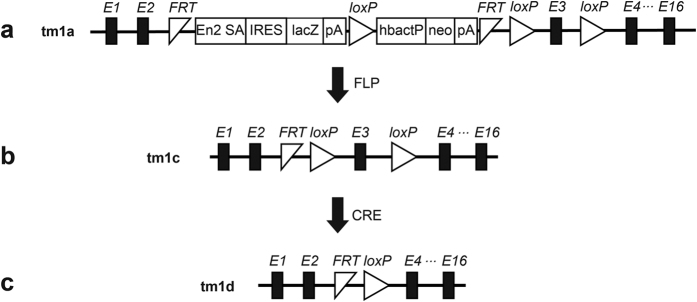
Schematic diagram of *Ampd1*^*tm1(KOMP)Wtsi*^ allele. (**a**) The *Ampd1* tm1a allele was initially a non-expressive form due to the alternative splicing of *Ampd1* exon1-2 to En2 SA in the trapping cassette, disrupting the *Ampd1* transcript. (**b**) *Ampd1* tm1c allele was produced by crossing *Ampd1* tm1a allele with an Flp transgenic mouse strain, removing the FRT-flanked knockout-first cassette. (**c**) *Ampd1* tm1d allele was generated by mating *Ampd1* tm1c allele with a Cre transgenic mouse strain, removing the exon 3 of Ampd1. E1…16, exons of *Ampd1*; En2 SA, mouse En2 splicing acceptor; IRES, internal ribozyme entry site; lacZ, lair conditionerZ; pA, poly A; hbactP, human beta actin promoter; neo, promoter-driven neomycin resistant gene; FRT, flippase recognition target; Loxp, locus of crossover in p1.

**Figure 2:**
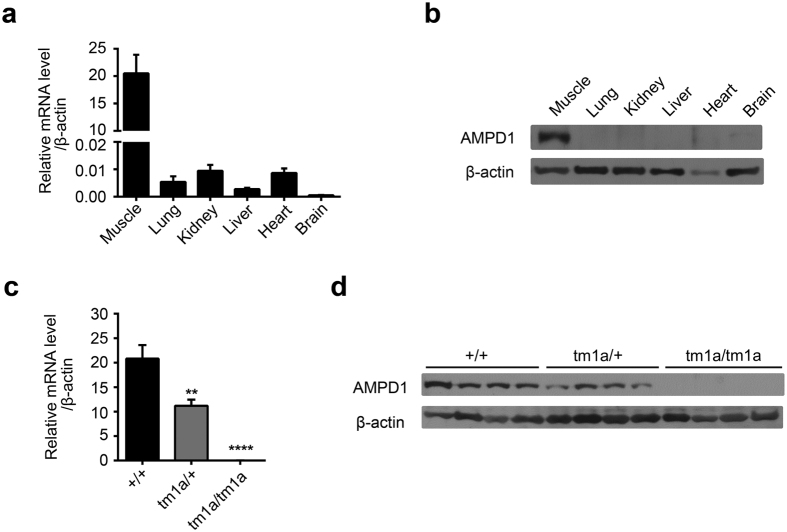
The expression of *Ampd1* in different tissues and the validation of *Ampd1* disruption. (**a,b**) The expression of *Ampd1* in different tissues. *Ampd1* was predominantly expressed in the muscle as determined with real-time PCR (**a**) and Western blot (**b**) in E18.5 WT mice. (**c,d**) The validation of *Ampd1* disruption. *Ampd1* mRNA (as compared with WT, ****P < 0.0001; **P < 0.001, One-way ANNOVA followed with Bonferroni’s multiple comparisons test) and protein (**d**) were undetectable in the hind-leg muscle of E18.5 *Ampd1*^*tm1a/tm1a*^ mice. Relative mRNA level was normalized to β-actin.

**Figure 3:**
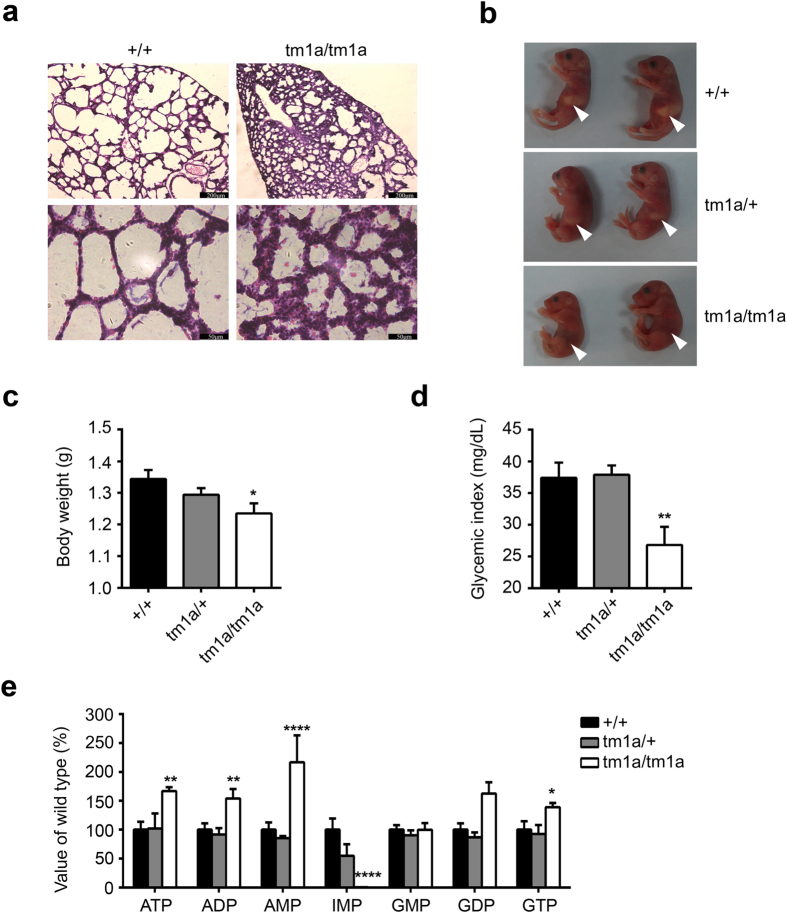
Phenotypic analysis of *Ampd1*^*tm1a/tm1a*^ mice. (**a**) H&E staining of the lung from P0.5 mice. *Ampd*1^tm1a**/**tm1a^ mice showed volume reduced pulmonary alveoli and incrassated alveolar septum compared to WT mice. Scale bar: 200 μm (upper), 50 μm (bottom). 7/7 mice showed this phenotype. (**b**) Representative P0.5 mice. No milk in *Ampd1*^*tm1a**/**tm1a*^ stomachs (arrowheads). (**c**) Reduced body weight of P0.5 *Ampd1*^*tm1a**/**tm1a*^ pups (WT: 1.343 ± 0.028 g, n = 15; *Ampd1*^*tm1a/*+^: 1.294 ± 0.021 g, n = 35; *Ampd1*^*tm1a/tm1a*^: 1.235 ± 0.031 g, n = 17; mean ± SEM. *P < 0.05, One-way ANNOVA followed with Bonferroni’s multiple comparisons test). (**d**) Decreased glucose levels in P0.5 *Ampd1*^*tm1a**/**tm1a*^ pups (*Ampd1*^*tm1a/*+^: 37.89 ± 1.48 mg/dL, n = 35; WT: 37.4 ± 2.43 mg/dL, n = 15; *Ampd1*^*tm1a/tm1a*^: 28.76 ± 3.33 mg/dL, n = 17; mean ± SEM. **P < 0.01, One-way ANNOVA followed with Bonferroni’s multiple comparisons test). Glucose was measured using OneTouch UltraMini glucometer (LifeScan, Inc., Milpitas, CA, USA). (**e**) In the muscle from E18.5 animals, ATP, ADP, AMP, GDP levels were significantly increased 66%, 64%, 117%, 39% respectively and IMP levels were decreased to nearly zero in *Ampd1*^*tm1a**/**tm1a*^ mice (*P < 0.05, **P < 0.01, and ****P < 0.0001, two-way ANNOVA followed with Bonferroni’s multiple comparisons test).

**Figure 4:**
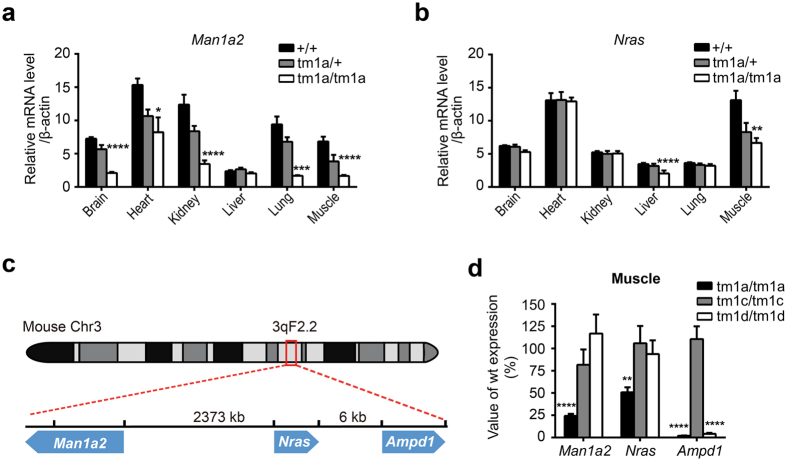
The knockout-first cassette in *Ampd1* tm1a allele influences the neighboring genes expression. (**a**) Real time PCR indicated that *Man1a2* was significant down-regulated in the brain, heart, kidney, lung and muscle; (**b**) *Nras* was slight down-regulated in the liver and the muscle of *Ampd1*^*tm1a/tm1a*^ mice (n = 5/each genotype. *P < 0.05, **P < 0.01, ***P < 0.001, ****P < 0.0001, two-way ANNOVA followed with Bonferroni’s multiple comparisons test). (**c**) Schematic diagram of *Man1a2, Nras* and *Ampd1* on chromosome 3. *Nras* is 6 kb from *Ampd1* gene, whereas *Man1a2* is 2.4 Mb away from *Ampd1*on the other strand. Arrowheads indicate orientation (left, negative strand; right, positive strand). (**d**) Relative mRNA expression of *Ampd1, Man1a2, Nras* in the muscle from different mice allele (As compared to WT, n = 5/each genotype, **P < 0.01, ****P < 0.0001, one-way ANNOVA followed with Bonferroni’s multiple comparisons test).

The *Ampd1*^*tm1a/*+^ mice (#CSD23933) were generated by KOMP. A cassette containing mouse En2 SA, LacZ, Neo, FRT and loxP sites, was inserted in introns around exon3 of *Ampd1* gene (Fig. 1a). The tm1a allele was initially an *Ampd1* non-expressive form. *Ampd1*^*tm1a/*+^ mice were obtained from the KOMP repository and intercrossed to generate *Ampd1*^t*m1a/tm1a*^. *Ampd1*^*tm1c/*+^ allele was produced by crossing *Ampd1*^*tm1a/*+^ with a globe Flp transgenic mouse strain [Gt(ROSA)26Sortm1(FLP1)Dym/J, The Jackson Laboratory Stock No:003946]. *Ampd1*^*tm1d/*+^ allele was generated by mating *Ampd1*^*tm1c/tm1c*^ with a globe Cre transgenic mouse strain [B6.C-Tg(CMV-cre)1Cgn/J, The Jackson Laboratory Stock No: 006054]. PCR primers used for genotyping were listed in Supplementary Table 2. All animal experiments were approved by the ethics committee of State Key Laboratory of Medical Genetics, Central South University of China. All methods were performed in accordance with approved guidelines.

### RNA extract and quantitative real-time PCR analysis

Total RNA were extracted from the hindleg muscle of E18.5 mice using Trizol reagent (Life technologies, NY, USA). cDNA was synthesized from total RNA (1 μg) through the RevertAid First Strand cDNA Synthesis Kit (K1622, Thermo Scientific, Inc., Waltham, MA, USA) and oligo dT primers. Quantitative real-time PCR was carried out with a CFX96 Touch™ Real-Time PCR Detection System (Bio-Rad Laboratories, lnc. Hercules, CA, USA) using Maxima SYBR Green qPCR Master Mixes (K0251, Thermo Scientific, Inc., Waltham, MA, USA). Data were normalized to β-actin and analyzed by the comparative cycle threshold (CT) method. Specific primers for each gene were as follows: 5′CAGAGCCTCGCTTATCCATC3′ and 5′TCTTGGATCGGAACACATCA3′ for *Ampd1*; 5′CACATCAGTTGGTGGTCTGG3′ and 5′GCCTCAACAGCATCGTCATA3′ for *Man1a2*; 5′TGACTTGCCAACAAGGACAG3′ and 5′CTGGCGTATCTCCCTTACCA3′ for *Nras*; 5′CACGATGGAGGGGCCGGACTCATC3′ and 5′TAAAGACCTCTATGCCAACACAGT3′ for *β-actin*.

### Immunoblotting

Mouse tissues were cut into pieces and added with 2xSDS buffer (63 mM Tris-HCl, 10% Glycerol, 2% SDS, 0.0025% Bromophenol Blue, pH 6.8) supplemented with protease inhibitor cocktails (P8430, Sigma-Aldrich, St. Louis, MO, USA). After ultrasonication, the lysate was centrifuged at 13,000 rpm for 10 min. The protein concentration of the supernatant was measured using the Pierce BCA^TM^ protein assay kit (23228, Thermo Scientific, Inc., Waltham, MA, USA). For western blot 40 μg proteins were separated in 10% SDS-PAGE gels and transferred to PVDF membrane, blocked with 5% milk in 0.1% PBS-T (0.1% Triton-X 100 in 1 × PBS), and blotted with primary antibody overnight at 4 °C. The antibodies for AMPD1 (sc-160043, Santa Cruz Biotechnology, Inc., Santa Cruz, CA, USA) and β-actin (A2228, Sigma-Aldrich, St. Louis, MO, USA) were purchased. The secondary antibodies were obtained from Jackson ImmunoResearch Laboratories (Jackson ImmunoResearch Laboratories, Inc., West Grove, PA, USA). ECL plus Western blotting detection system (GE Healthcare, Chalfont St. Giles, Buckinghamshire, UK) was used to detect immobilized specific antigens, according to manufacturer’s instruction.

### Histological examination

Tissues were fixed in 4% paraformaldehyde, following dehydration with sucrose, tissues were frozen immediately in precooling isopentane, and sectioned to 15 μm with Leica CM1950 freezing microtome (Leica Biosystems, Wetzlar, Germany). Sections were stained with Harris’s hematoxylin and eosin (H&E). Images were taken using Leica DM5000B Microscope System (Leica Microsystems, Wetzlar, Germany).

### Nucleotide analysis by High Performance Liquid Chromatography (HPLC)

Fresh-frozen tissues were washed once with PBS, and homogenized in 10% Trichloroacetic Acid (T6399, Sigma-Aldrich, St. Louis, MO, USA). After incubating on ice for 10 min, the mixtures were centrifuged at 12,000 g for 15 min at 4 °C, and the nucleotide containing supernatant was washed 4 times with water-saturated diethyl ether. Then the supernatant was froze at −80 °C, and freeze-dried at −20 °C overnight use FreeZone Freeze Dry Systems (LABCONCO, Inc., MO, USA). The extracted nucleotides were dissolved in 100 μL ultrapure water and centrifuged at 8,000 rpm for 5 min. Supernatant was filtered with 0.45 μm filter and then analyzed by HPLC on a Agilent 1260 (Agilent Technologies, Inc., CA, USA) equipped with a Waters SunFireTM C18 column (250 mm × 4.6 mm i.d., 5 μm, Waters, Milford, MA, USA) and detected by UV-VIS absorbance at 260 nm. The nucleotides were eluted with isocratic elution (flow rate: 0.75 ml/min, Column temperature: 25 °C) using a mixture of 10% acetonitrile and 90% water (0. 01 mol/L Tetrabutylammonium bromide, 0.05 mol/L Na_2_HPO_4_-NaH_2_PO_4_, pH6.4). The amount of each nucleotide was determined by external standard method. The following standards were used: ATP (A2383), ADP (A2754), AMP (01930), IMP (57510), GMP (G8377), GDP(G7127), GTP(G8877) (Sigma-Aldrich, St. Louis, MO, USA).

### RNA sequencing (RNA-seq)

RNA sequencing was performed by Novogene Bioinformatics Technology Co. Ltd. The RNA was extracted from the hindleg muscles of embryonic 18.5 day mice (3 WT and 3 *Ampd1*^*tm1a/tm1a*^) by using Trizol reagent (Life technologies, NY, USA). A total of 3 μg RNA per sample was used. Sequencing libraries were generated using NEBNext^®^ Ultra™ RNA Library Prep Kit for Illumina^®^ (New England Biolabs., MA, USA) following manufacturer’s recommendations and index codes were added to attribute sequences to each sample. The clustering of the index-coded samples was performed on a cBot Cluster Generation System using TruSeq PE Cluster Kit v3-cBot-HS (Illumina, San Diego, CA, USA) according to the manufacturer’s instructions. After cluster generation, the library preparations were sequenced on an Illumina Hiseq 2000 platform and 100 bp single-end reads were generated.

### RNA-Seq data analysis

Raw data were firstly processed to obtain clean data by removing reads containing adapter, ploy-N and low quality reads. Index of the reference genome was built using Bowtie v2.0.6 and single-end clean reads were aligned to the reference genome using TopHat v2.0.9. And HTSeq v0.5.4p3 was used to count the reads numbers mapped to each gene. Then RPKM (Reads Per Kilobase of exon model per Million mapped reads) of each gene was calculated based on the length of the gene and reads count mapped to this gene. Differential expression analysis was performed using the DESeq R package (1.10.1). The resulting P-values were adjusted using the Benjamini and Hochberg’s approach for controlling the false discovery rate. Genes with an adjusted P-value < 0.05 found by DESeq were assigned as differentially expressed.

### Statistical analysis

All statistical analyses were performed using the Prism 6.01 (Graph Pad Software, San Diego, CA). Statistically significant differences between groups were determined by a repeated ANOVA followed by unpaired *t* test.

## Additional Information

**How to cite this article**: Pan, Y. *et al*. Insertion of a knockout-first cassette in *Ampd1* gene leads to neonatal death by disruption of neighboring genes expression. *Sci. Rep.* **6**, 35970; doi: 10.1038/srep35970 (2016).

## Supplementary Material

Supplementary Information

## Figures and Tables

**Table 1:** The distribution of progeny from mating of heterozygotes with different *Ampd1* alleles.

Allele	Stage	WT: Heterozygotes: Homozygotes	
		Total Number	Ratio
tm1a	*E18.5	184	1:1.98:0.94
	#P0.5	126	1:2.09:0.84
	P1	112	1:2.07:0.67
	P2	89	1:2.07:0
	Adult	407	1:1.81:0
tm1c	Adult	69	1:2.17:0.88
tm1d	Adult	62	1:2.06:0.875

^*^E: embryonic.

^#^P: postnatal.
