# Supplementary material for: Insertion of a knockout-first cassette in Ampd1 gene leads to neonatal death by disruption of neighboring genes expression
Source: Sci Rep. 2016 Oct 24;6:35970. doi: 10.1038/srep35970 (PMC5075929; doi:10.1038/srep35970)
Supplement: Supplementary Information [file srep35970-s1.pdf]

Insertion of a knockout-first cassette in *Ampd1* gene leads to neonatal death by disruption of neighboring genes expression

Yongcheng Pan[1,2#], Lusi Zhang[1,3#], Qiong Liu[1], Ying Li[1], Hui Guo[1], Yu Peng[1], Hexiang Peng[1],Beisha Tang[1,2,4], Zhengmao Hu[1], Jingping Zhao[5], Kun Xia[1, 6, 7]*, Jia-Da Li[1,7]*

Supplementary Information

Supplementary Table 1: Differentially expressed genes identified by RNA sequencing and RT-PCR verification.

Gene	Chr/Strand	Start/End	RNA seq		RT-PCR	
			Value of WT	p-adjust	Value of WT	p-value
Man1a2	3/-	100562208/100685503	18.07% ±2.30%	9.070E-53	24.37% ±2.19%	0.0001
Ptgfrn	3/-	101040232/101110278	100.33% ±5.80%	1	107.37% ±23.93%	0.7776
Igsf3	3/+	101377125/101463060	101.78% ±4.13%	1	103.57% ±19.44%	0.8723
Atp1a1	3/-	101576219/101604603	113.65% ±0.79%	1	115.33% ±14.01%	0.4038
Casq2	3/+	102086415/102146514	84.78% ±7.41%	1	80.17% ±16.35%	0.4416
Csde1	3/+	103020546/103058189	101.64% ±7.75%	1	86.48% ±10.34%	0.1547
Nras	3/+	103058339/103067914	63.63% ±5.43%	3.314E-04	50.85% ±5.53%	0.0037
Ampd1	3/+	103074014/103099714	0.16% ±0.04%	6.089E-38	0.11% ±0.16%	0.0003

In RNA sequencing, muscle tissue come from E18.5 WT mice (n=3) and *Ampd1*$^{tm1a/tm1a}$ mice (n=3). The P-values were adjusted using the Benjamini and Hochberg's approach for controlling the false discovery rate. Another group of E18.5 WT mice (n=5) and *Ampd1*$^{tm1a/tm1a}$ mice (n=5) muscle were used for RT-PCR. Two-tailed Student's *t* test was used to determine the significance of differences, mean ± SEM.

Supplementary Table 2: Genotyping primers and PCR products size of different *Ampd1* mice.

Allele	Primer	Sequence(5'-3')	Product size(bp)
Ampd1^{tm1a}	WT-F	tgggctgacagctgcagttagca	WT:354bp<br>tm1a/+:354bp & 410bp<br>tm1a/tm1a:410bp
	loxP-F	gagatggcgcaacgcaattaat	
	Tm1a-R	ccaagaaaaagacagcacaggagacc	
Ampd1^{tm1c}	Tm1c-F:	ggcaactcccttttaccaca	WT:352bp<br>tm1c/+:516bp & 352bp<br>tm1c/tm1c:516bp
	Tm1c-R	tttccctgatctccaaccag	
Ampd1^{tm1d}	Tm1d-F	ggcaactcccttttaccaca	WT:1090bp<br>tm1d/+:1090bp & 400bp<br>tm1d/tm1d:400bp
	Tm1d-R	ggtaacacgcatgacaggtg	

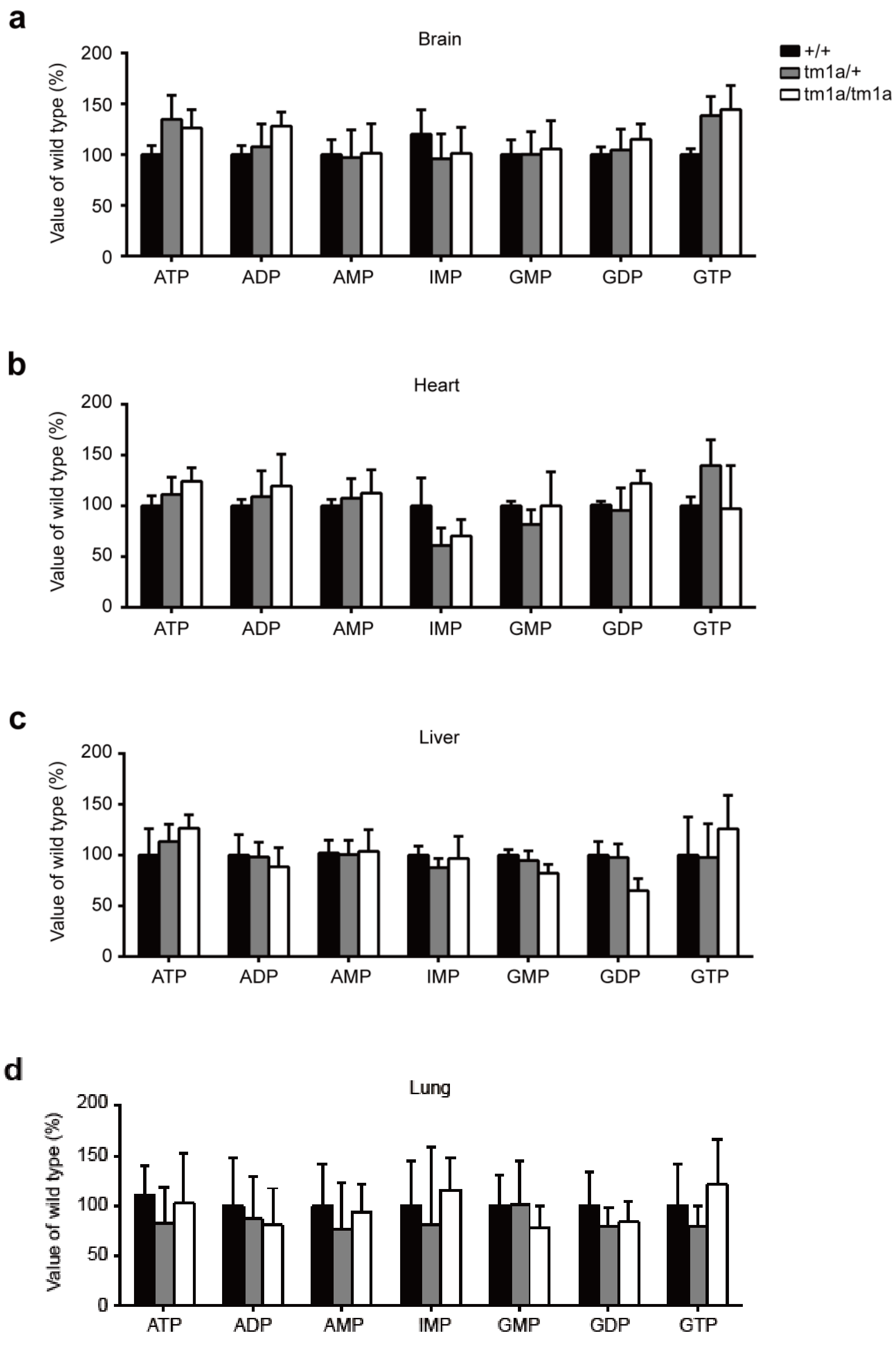

Supplementary Figure 1: Nucleotide levels in the brain, the heart and the liver of E18.5 mice. There is no genotypic difference in the nucleotide levels of the brain (a), the heart (b), the liver (c) and the lung (d) in E18.5 *Ampd1*$^{tm1a/tm1a}$ mice. n=5/each genotype, mean ± SEM.

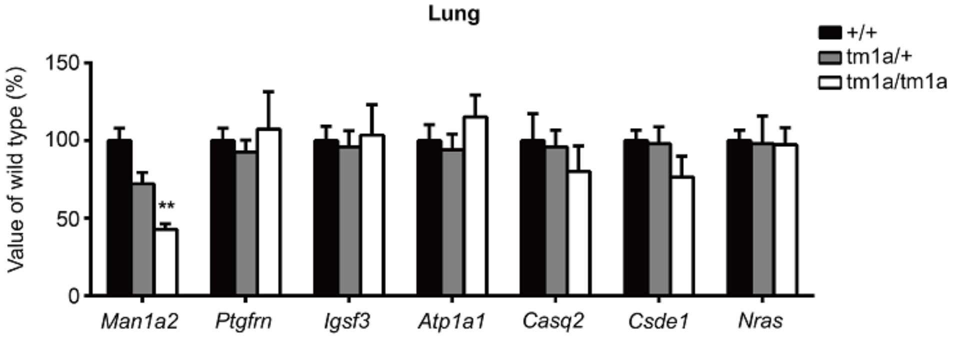

Supplementary Figure 2: RNA level of different genes, which located between *Ampd1* and *Man1a2,* in the lung of *Ampd1*$^{tm1a/tm1a}$ mice**.** Man1a2 expression level was significantly decreased to 42.69%±3.52% in lung of *Ampd1*$^{tm1a/tm1a}$ mice, but the expression of genes located between *Man1a2* and *Ampd1* (*Ptgfrn, lgsf3, Atp1a2, Casq2, Csde1* and *Nras*) did not change in lung (n=5/each genotype, mean ±SEM. As compared with WT, **P<0.01, two-way ANNOVA followed with Bonferroni's multiple comparisons test).

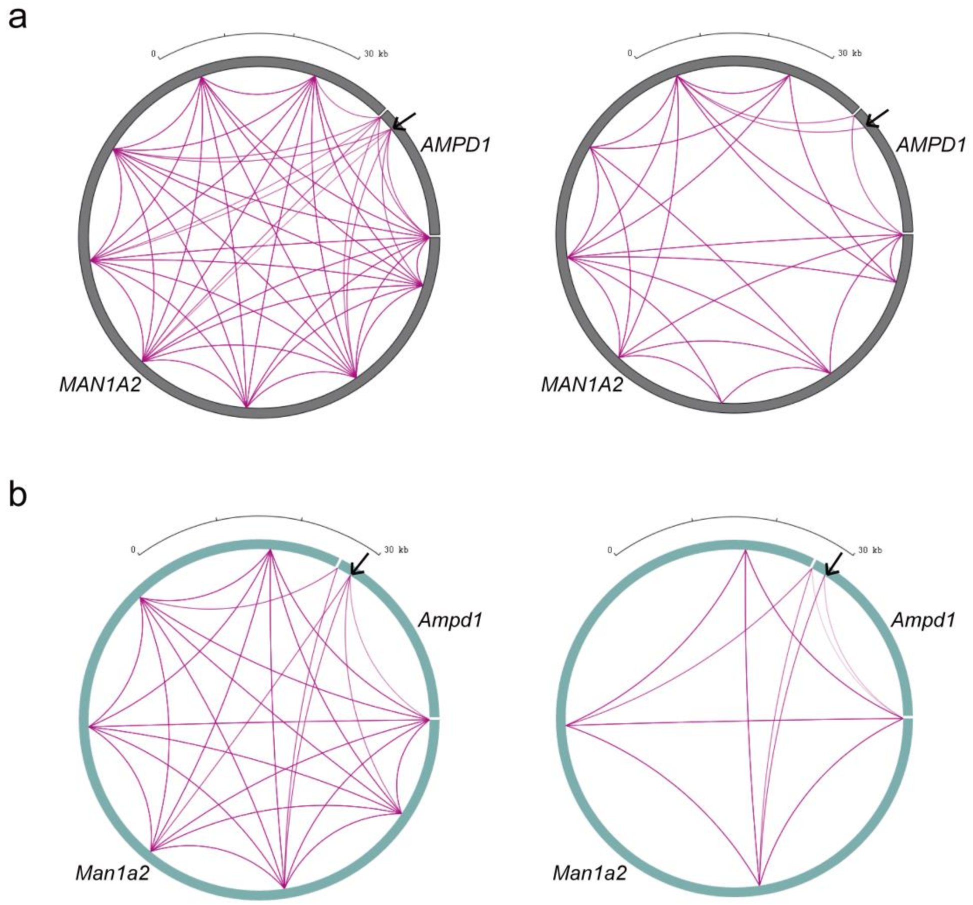

Supplementary Figure 3: Potential long range interactions between *Ampd1* and *Man1a2* gene. Hi-C results hosted in WashU Epigenome Browser indicated probable long range interaction between *Ampd1* and *Man1a2* in (a) human H1 embryonic stem cell or (b) mouse embryonic stem cell. Purple arc represented Long range interaction.
